# Redefining Success in Hernia Surgery: The Case for Patient-Reported Outcomes

**DOI:** 10.3390/jcm14176131

**Published:** 2025-08-29

**Authors:** Jacob Rosenberg, Anders Gram-Hanssen, Hugin Reistrup, Jason Joe Baker

**Affiliations:** Center for Perioperative Optimization, Department of Surgery, Herlev and Gentofte Hospital, University of Copenhagen, 2730 Herlev, Denmark; anders.gram-hanssen@regionh.dk (A.G.-H.); hugin.reistrup.01@regionh.dk (H.R.); jason.joe.baker@regionh.dk (J.J.B.)

**Keywords:** patient-reported outcomes, hernia repair, benign surgery, surgical evaluation, quality of life, patient satisfaction

## Abstract

In elective hernia surgery, the primary aim is to improve quality of life, rather than to save life. Therefore, outcome measures should emphasize domains such as pain, function, and overall satisfaction. While some principles also apply to other benign procedures, this perspective article centers on hernia repair as a paradigm for redefining surgical success. We perform hernia surgeries primarily due to quality-of-life concerns, and, consequently, it makes sense that outcome measures should emphasize quality-of-life indicators such as pain, other complaints impacting daily life, and most importantly, overall patient satisfaction with the procedure. Nonetheless, many interventional studies related to hernia disease tend to focus on tangible surgical outcomes like recurrence, infections, hospital stays, and readmissions. Therefore, we advocate for a shift in the evaluation of surgeries to prioritize more relevant patient-reported outcomes when assessing the effects of surgical procedures for benign conditions. These considerations not only apply to hernia surgery but also to other surgical interventions where the indication for surgery is based on quality-of-life issues. We urge the systematic incorporation of patient-reported outcomes into surgical practices and outcomes research to promote a more patient-centered approach, aligning surgical success with the outcomes that matter most to patients.

## 1. Introduction

Surgical procedures are often evaluated using traditional, disease-specific endpoints that may not capture patient-centered outcomes. Unlike in malignant surgery, where cure and survival are the primary endpoints, hernia surgery is performed mainly to improve quality of life. In general, most benign surgeries aim to enhance a patient’s quality of life rather than extend it. Therefore, the goals and results of these procedures should prioritize quality-of-life improvements such as pain relief, reduced discomfort, and overall satisfaction with the procedure [1]. The preoperative complaint that prompted the surgical procedure should be improved post-surgery, so why not directly ask if this goal was achieved? Unfortunately, the assessment of benign surgical outcomes in current literature is largely guided by traditional clinical parameters (e.g., recurrence or infection rates) and hospital length of stay, which often do not fully reflect what matters most to patients.

In hernia disease, typical outcome parameters reported in the literature include recurrence, re-operation for recurrence, and both acute and chronic pain. However, suppose a male patient with a large bulge in the inguinal region cannot have sexual intercourse with his partner due to the physical presence of the bulge. In that case, he may wish to undergo groin hernia surgery to improve this situation. Perhaps after the operation, he can enjoy normal sexual function with his partner, but he develops a small hernia recurrence and may even experience slight occasional chronic pain after the procedure. Under traditional hernia literature standards, his outcome would be considered a failure due to both recurrence and chronic pain. Nevertheless, he may feel very satisfied overall with the procedure because the main issue of being able to have intercourse with his partner has been resolved. This example underscores the importance of measuring outcomes that matter most to patients, rather than focusing solely on traditional surgical outcome parameters. Similarly, a patient undergoing ventral hernia repair may be primarily concerned about resuming heavy lifting at work. Even if a small seroma develops postoperatively—a complication under traditional metrics—successful return to work within weeks may represent a positive outcome for that individual.

However, while hernias are generally benign, it is obviously important to acknowledge that emergencies (e.g., incarceration) may occur. The annual risk of incarceration for patients with incisional hernias is <3% [2], and for patients with groin hernias, the annual risk is <1% [3]. Such events may result in small bowel obstruction—of which hernias are a common cause—and necessitate emergency surgery. Given the increased morbidity and mortality associated with emergency repair, elective repair is generally preferable when the patient’s risk profile and symptom burden justify intervention. Under these circumstances (i.e., emergency hernia repair), the criteria of success are obviously significantly different from in an elective setting. This perspective article is mainly focused on elective surgery and its outcome measurement.

This perspective article argues that patient-reported outcomes (PROs) should be adopted as the primary benchmark of success in hernia surgery, complementing traditional measures. PROs are measures derived directly from patients about their health status, quality of life, and symptom burden without interpretation by clinicians. While some parts of the discussion in this perspective article relate to hernia surgery, the principles apply to any benign procedure.

Despite the inherent limitations of PROs, they provide a patient-centered framework for evaluating surgical care in non-life-threatening conditions, explicitly aiming for improved health and satisfaction over time as experienced by those undergoing these operations. Hence, this paper seeks to promote a new paradigm in assessment and reporting by prospectively integrating PROs into the overall evaluation process of outcomes after various benign surgical procedures. Traditional outcome metrics—such as recurrence, readmission, and length of stay—do not fully reflect patients’ priorities in hernia surgery. We propose integrating PROs as complementary primary endpoints to capture the success of surgery from the patient’s perspective. This perspective article focuses primarily on hernia surgery as the anchor for discussion, while drawing brief parallels to other benign procedures where appropriate. The discussion first reviews the limitations of traditional outcomes, then highlights the disconnect with patient experience, introduces validated PRO tools with emphasis on hernia surgery, and finally explores practical implementation and future directions.

## 2. The Traditional Focus on Measurable Clinical Outcomes like Recurrence and Readmissions

The success of hernia surgery has traditionally been assessed using various clinical outcomes, including recurrence rates, infections, and readmissions [4,5,6]. While these measures provide a straightforward and objective way to evaluate the technical success of surgery, they often overlook PROs.

All these traditional outcome measures offer the advantage of quantifiable data that can be easily compared against benchmarks and across different surgical techniques. They standardize metrics such as complication rates, recurrence rates, and readmissions, enabling comparisons between institutions and individual surgeons. These measures persist because they are easy to collect, they are objective, embedded in national registries, and linked to reimbursement and quality reporting frameworks. These measures are essential for providing surgical teams with feedback to improve their protocols and enhance patient safety, and they may also guide treatment guidelines. However, they might not fully capture outcomes directly related to the primary reasons for surgery, like pain relief or quality of life improvements affected by the benign condition.

For example, while a recent study demonstrated the technical success of robotic surgery, showing lower short-term complication rates and better recovery scores after laparoscopic or open procedures [7], these metrics do not necessarily correlate with increased patient satisfaction or overall quality of life in long-term follow-ups. Another study found that while complication rates and readmission rates are important metrics, they may not fully capture the long-term experiences and functional outcomes of patients undergoing shoulder replacement surgery [8].

## 3. Disconnect Between Clinical Outcomes and Patient Experience

We have extensive data indicating that patient satisfaction and perceptions of quality-of-life improvements after surgery can vary greatly from our objective clinical measures. A systematic review of orthognathic surgery outcomes reported that these treatments often result in significant quality-of-life enhancements. However, many of these improvements are not captured by traditional clinical outcome metrics [9]. The review included 30 studies that assessed quality of life and patient satisfaction through various questionnaires. They found that clinical outcomes, such as dental and facial harmony, were generally good. Nonetheless, patient satisfaction remained low, highlighting the need to develop better tools for measuring patient-reported outcomes to more effectively align with patients’ expectations for disease management.

A study on instruments to measure patient satisfaction in spine surgery found that current tools lack the detail and specificity needed for clinical use [10]. They observed that patient satisfaction is most often assessed as a single, overall measure rather than as a multidimensional one, with ratings mainly influenced by clinical factors instead of psychosocial or functional aspects. The study identified seven dimensions that affect patient satisfaction: pain, function, patient expectations/preferences, specific health conditions (co-existing diseases), caregiver interactions with patients, and the effectiveness of postoperative care/therapy—highlighting the need for a disease-specific, comprehensive tool. This gap results in limited understanding of the patient’s overall experience and underscores the importance of incorporating patient-reported outcomes into standard assessments.

A concurrent analysis of patient satisfaction after inguinal hernia repair highlights the gap between clinical outcomes and patient-reported experiences [11]. Their review of existing literature found that, although patient satisfaction generally appeared positive, the measures used were often unvalidated and inconsistent. The methodological differences across studies and the reliance on skewed response questionnaires led to notable disparities in reported satisfaction rates. This inconsistency underscores the critical need for standardized, validated tools that can accurately measure patient satisfaction and offer a clearer picture of surgical success.

A systematic review evaluated the level of evidence supporting the measurement properties of patient-reported outcome measures (PROMs) used to assess recovery after general abdominal surgery [12]. The study included 22 different PROMs and found that half of them were only supported by “limited” or “unknown” levels of evidence. Additionally, the study concluded that none of the included PROMs could be recommended for use because of generally insufficient evidence regarding their measurement properties. In other words, all 22 PROMs were inadequately validated. A similar systematic review looked specifically at PROMs designed to evaluate postoperative outcomes after groin hernia surgery [13]. This review included 11 PROMs and found that none were sufficiently validated; therefore, they could not be recommended. A common issue in these two reviews is the lack of evidence for the content validity of the included PROMs. Adequate content validity requires evidence that a PROM measures what it is supposed to measure—what matters most to patients [14]. This is problematic because it means we cannot be certain that we are truly asking patients the right questions or addressing the issues they consider most important. Other general PROM issues include limited responsiveness to change, ceiling effects, and variation in scoring, all of which undermining comparability across studies. Improving PROMs requires involving patients in item generation, conducting rigorous psychometric testing, and ensuring cultural adaptation for international use. Across diverse surgical fields, a consistent pattern emerges: validated, disease-specific tools are scarce, and traditional metrics rarely align with patient satisfaction.

## 4. The Rise of Patient-Reported Outcomes in Surgical Evaluation

Recently, the field of surgery has experienced a growing interest in PROs, reflecting a shift toward more patient-centered care. PROs include various metrics collected directly from patients that indicate their health status, quality of life, and symptom burden, as well as their perspectives on the effectiveness (benefits) or tolerability (burdens) of treatments. Unlike traditional metrics, which reflect clinical processes and complications, PROs capture the patient’s subjective evaluation of symptom resolution and functional recovery over time. These outcomes serve as practical tools for measuring the impact of surgical interventions in patient-centered analyses. An example is a study involving breast cancer patients who underwent free-flap reconstruction after mastectomy. They used the BREAST-Q questionnaire [15], a validated instrument for assessing different aspects of satisfaction, including aesthetic satisfaction, psychosocial well-being, and sexual function. The research team concluded that patient satisfaction with breast reconstruction outcomes improved over time. Specifically, patients who had reconstruction more than five years earlier reported significantly higher satisfaction compared to those who had surgery within the last five years. This underscores the importance of tracking PROs over an extended period to better understand recovery pathways for both patients and healthcare providers, helping to establish realistic expectations.

Probably the largest and most comprehensive project using this modern approach to outcomes assessment in benign surgery is the AFTERHERNIA Project [16]. The AFTERHERNIA Project is a nationwide, population-based survey using registry linkage to identify all patients undergoing groin or ventral hernia repair in Denmark since 2014. It systematically measures PROs alongside traditional outcomes, allowing granular comparisons of surgical techniques and patient subgroups. As of 2025, it is the biggest investigation into how patients fare after hernia surgery, measuring multiple parameters, including relevant PROs based on nationwide data that covers all patients who have undergone hernia surgery in the past decade. This is possible because of Denmark’s nearly fully digital infrastructure, where 95% of adult Danes have access to Digital Post, greatly simplifying communication with a large portion of the population. This advantage is further strengthened by the fact that Danish healthcare is completely register-based, effectively turning the entire country into a cohort [17]. The AFTERHERNIA Project will give clinicians worldwide insights into the effects of different surgical techniques on relevant PROs, helping to facilitate evidence-based clinical decisions—based not on outcomes defined by the surgeon, but on those determined by the patient. The first results from this ambitious project are expected in 2025/2026. While Denmark’s digital infrastructure facilitates large-scale PRO collection, countries without similar systems may require phased implementation using smaller regional registries or mobile platforms.

To advance the field, it is important to establish standardized practices for including PROs in both research and clinical care pathways. Validated tools, such as the BREAST-Q [15], Abdominal Hernia-Q [18], and EORTC instruments [19], can simplify data collection and interpretation, enabling large-scale implementation. Understanding patient experiences through PROs is vital for improving care quality and increasing patient satisfaction. In clinical research, PROs can help identify the best treatments for patients.

## 5. Examples of Patient-Centered Outcomes in Benign Surgical Procedures

**Figure 1:**
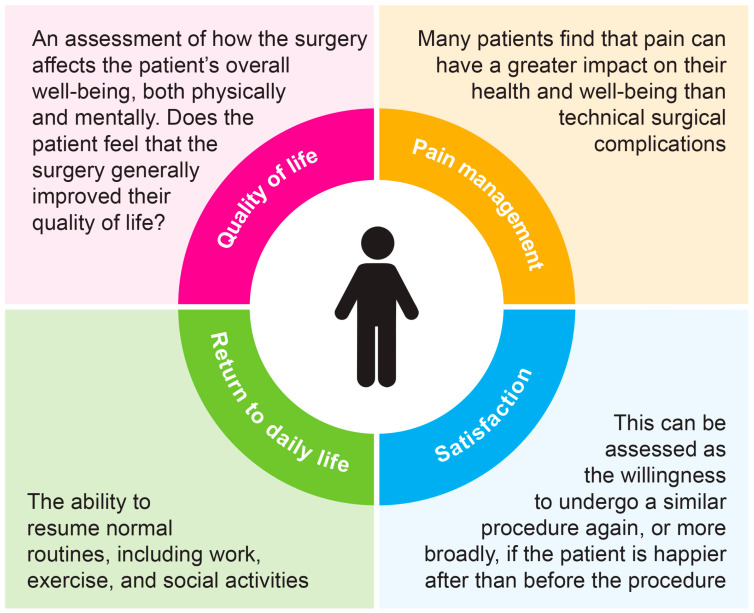
Essential patient-reported outcomes in benign surgery.

The patient-centered outcomes such as quality of life, pain management, and functional recovery are essential for assessing the success of benign surgical procedures. Including PROs in surgical evaluations ensures a comprehensive review, emphasizing the main goal of surgery: enhancing patients’ well-being. Important patient-reported outcomes in benign surgery may vary depending on the procedure, but generally include quality of life, pain control, return to daily activities, and overall satisfaction (Figure 1).

An interesting new approach to patient-centered monitoring of surgical outcomes is the use of the Measure Yourself Medical Outcome Profile (MYMOP) [20]. In this approach, the patient is asked what their main problem is before an intervention and then, in follow-up, if the intervention has improved that specific parameter. This is exactly what we want to achieve as surgeons—to improve the specific parameter that led the patient to surgery.

## 6. Standardized Questionnaires and Valid Measurement Tools for PROs

Implementing PROs into clinical practice, especially in benign surgery, requires the use of standardized questionnaires and reliable measurement tools. These instruments are important for collecting accurate, relevant, and actionable data about patient experiences, quality of life, and overall satisfaction with surgical procedures.

Standardized questionnaires are carefully designed to include a set of validated and consistent questions that patients answer regarding their symptoms, functional status, and overall well-being. These questionnaires can be used in both research settings and routine care to ensure systematic data collection. These PRO instruments are essential for personalizing patient care by interpreting and responding to individual PRO scores [21]. It is advisable to use both a generic and a disease-specific instrument for evaluating PROs after surgery. For example, the Medical Outcomes Study 36-Item Short-Form Health Survey (SF-36) [22] is often used to assess general health status, while disease-specific tools, such as the Western Ontario and McMaster Universities Osteoarthritis Index (WOMAC) [23], focus on particular conditions. Several other well-validated instruments tailored to benign surgical conditions are available, including the Abdominal Hernia-Q for ventral hernia surgery [18], the Oxford Hip Score for hip replacement surgery [24], and the Aberdeen Varicose Vein Questionnaire for varicose vein surgery [25]. The goal is to develop a comprehensive understanding of patient outcomes that goes beyond traditional clinical metrics.

Another vital aspect is ensuring that these questionnaires and tools are validated across diverse populations to maintain their reliability and effectiveness. For example, the International Consortium for Health Outcomes Measurement (ICHOM) emphasized the importance of creating a standard set of outcomes for patients with congenital heart disease, including both clinical and patient-reported outcomes [26]. They developed a 15-item outcome set through a comprehensive literature review, multiple-round Delphi processes, and stakeholder consultations. This thorough approach guarantees that the selected outcomes are meaningful and applicable across various patient groups and clinical settings. By involving different stakeholders, including patients, this initiative ensures that the tools are relevant and user-friendly, increasing their usefulness in routine clinical practice. Initiatives in hernia surgery have also been launched with the development of core outcome sets in groin [27] and incisional [28] hernia repair.

However, implementing these standardized tools in daily clinical practice may present its challenges. There is high demand for resources and time, which can strain healthcare providers. Additionally, the complexities of data analysis create a significant barrier. In daily practice, integration of PROs into surgical workflows typically begins with a baseline preoperative assessment, followed by structured postoperative follow-up at defined intervals. These data are then linked to existing registries or electronic health records, enabling comparison across patients and institutions. Finally, aggregated feedback can be provided to clinicians and patients to guide shared decision-making and continuous quality improvement. Clinicians and administrative staff require proper training and support to effectively integrate PRO measures into their practice. This includes understanding the technical aspects of data collection, interpretation, and how to incorporate them into clinical decision-making. Resources must be allocated for developing and maintaining electronic health record systems capable of storing and analyzing PRO data, and both clinicians and patients should understand PRO results and how to respond to them. Patient-centered questionnaires should be standardized and validated when including PROs in benign surgical practice, ideally as part of core outcome sets specific to the surgical procedure.

## 7. Potential Developments in Using Pros for Assessing Surgical Success

Recent advances highlight the growing importance of PROs in surgical evaluation. The Patient-Reported Outcomes Measurement Information System (PROMIS) shows significant promise, especially in spine surgery, where traditional metrics may not fully reflect patient well-being. A systematic review indicated that PROMIS strongly correlates with PRO measures, providing a reliable way to measure patient experiences post-surgery [29]. The review also noted that the use of PROMIS in spine research has steadily increased since 2012, although its main use remains in validation rather than routine outcomes measurement. This trend underscores the need to recognize the value of incorporating PROs and reveals a gap in clinical practice where further integration is needed.

The use of artificial intelligence (AI) in benign surgical conditions, like benign prostatic hyperplasia, further highlights this shift. AI has the capacity to transform diagnostics, management, and prognostication in benign surgery, emphasizing quality of life impacts that are sometimes overlooked in favor of malignant conditions [30]. It can help gather and analyze large amounts of PRO data, allowing for real-time adjustments and personalized care plans. When integrated with PROs, this technology can improve individualized patient care and provide a more comprehensive assessment of surgical success by considering both clinical and experiential factors. In the near future, AI is anticipated to be incorporated into electronic health record systems to continuously track PROs during procedures. This may, however, face challenges including technical limitations, ethical concerns, and uncertainty regarding autonomous actions [31]. Global disparities in access to technology further complicate implementation, as highlighted in a recent international cohort study on hernia surgery [32]. Nevertheless, advances are being made: AI is increasingly being explored for predicting outcomes and optimizing surgical performance in hernia repair [33], while recent reviews underscore both progress and persistent uncertainties in abdominal wall surgery [34].

AI use must address risks including algorithmic bias, opaque decision-making (‘black box’), and maintaining patient confidentiality when processing sensitive PRO data. Pilot programs in diverse clinical settings could evaluate feasibility, identify barriers, and refine workflows before wider rollout. An ideal evaluation would be a longitudinal, mixed-methods study with baseline PROs, repeated follow-ups, patient stratification, and linkage to clinical and economic outcomes. Clear regulatory frameworks will be needed to standardize AI–PRO integration, define data governance, and ensure interoperability with health IT systems.

It may also be possible to incorporate economic outcomes into the overall assessment of surgical success based on PROs. The Value-Based HealthCare (VBHC) model, as demonstrated in thoracic surgery, offers a progressive framework for evaluating surgical results through a combination of clinical and cost metrics, including key performance indicators (KPIs) that assess both clinical outcomes and costs on a seven-level Likert scale to determine value per patient [35]. The VBHC model has demonstrated that value delivery can improve even when costs increase, marking a significant advancement in outcome assessment. By applying a similar approach to benign surgical procedures, PROs could be systematically integrated to provide a more comprehensive view of surgical success, balancing patient satisfaction with clinical effectiveness and economic factors.

Implementing these advancements in clinical practice, however, presents some challenges. The operationalization of PROs requires standardized measures, such as the PROMIS domains [29], which can serve as benchmarks across various surgical fields. Furthermore, integrating AI demands significant investment in technology and training [30]. Healthcare facilities also need to consider ethical implications, ensuring patient privacy and data security as AI systems manage sensitive health information.

Furthermore, the VBHC approach requires a realignment of financial and operational strategies within healthcare institutions. Introducing VBHC in thoracic surgery led to clear improvements in patient care and operational efficiency [35], suggesting that a similar shift in benign surgeries could yield positive results. However, implementing this would need a significant reconfiguration of existing systems to effectively incorporate new KPIs.

Research will be important for optimizing PROs, AI, and VBHC in benign surgeries. Cross-disciplinary efforts and real-time data analysis will improve PRO use, leading to more patient-centered care and redefining surgical success by connecting clinical outcomes with patient experiences. The integration of PROs, fueled by technological innovations and value-based care, will make surgical evaluation more focused on patients.

There may be pressure from different stakeholders to implement multiple instruments simultaneously, but using multiple PROMs risks patient fatigue, reduced completion rates, and challenges for clinicians in interpreting multiple scores. Practical implementation requires clear assignment of responsibilities for data collection and funding. Hospitals can embed PRO collection into pre- and postoperative clinics, supported by nursing or administrative staff. Reimbursement models may incentivize reporting, for example, by tying part of payment to PRO submission. Public payers and insurers could mandate PRO reporting as a condition for coverage. Such strategies would make integration of PROs and AI both feasible and sustainable.

## 8. Research Opportunities for Measuring and Integrating Patient Experiences in Surgery

The use of PROs in surgical evaluation presents many research challenges aimed at improving patients’ emotional, psychological, and physical experiences. One key area is the validation of patient-reported experience measures (PREMs), where patients’ perceptions of their treatment by staff—such as respect and communication—are as important as clinical outcomes. Therefore, identifying sensitive PREMs can better inform healthcare practices, and PREMs may also influence certain PROMs. PREMs and qualitative interviews may complement PROMs to capture both outcomes and care experiences.

Another research opportunity exists in mixed-methods studies that combine qualitative and quantitative approaches to provide a comprehensive view of patient experiences after surgery. A recent mixed-methods survey following emergency laparotomy procedures revealed the complexities of patient recovery and life changes after the operation [36]. The study identified significant issues such as unmet needs in surgical aftercare, mental health support, and the necessity for timely restorative surgeries. Such research provides invaluable data that can help identify patient populations at a higher risk of negative outcomes and tailor interventions accordingly. The qualitative insights derived from patient feedback can be especially useful in shaping postoperative care guidelines, ensuring they align more closely with patient needs and expectations.

Using qualitative methods to understand each patient’s journey after surgery can also be very helpful. A study looked at patient experiences following sacrectomy, revealing different aspects of their post-surgical lives, such as chronic pain, the need for better information about long-term recovery, and the impact on family dynamics [37]. These findings highlight the transformative nature of major surgical procedures and emphasize the importance of thorough pre-operative counseling and strong, long-term follow-up care. By focusing on qualitative assessments, researchers can capture the unique and often overlooked parts of patient experiences, providing a story that enriches quantitative data.

Furthermore, using advanced data analytics and technology can improve how patient experiences are assessed. Digital health platforms and mobile apps can gather real-time PROs, offering ongoing feedback that tracks recovery progress and facilitates quick issue resolution. Incorporating these technology-driven approaches into everyday clinical practice opens an exciting path for future research, with the potential to make data collection and analysis easier, more accurate, and faster.

PROMs provide a unique chance to customize surgical care based on individual patient needs. They allow a deeper understanding of which surgical methods best match different patient preferences, baseline traits, and recovery goals. By emphasizing PROs in research on benign surgical conditions, it becomes possible to identify which treatment strategies produce the best outcomes for specific patient groups [16]. This supports evidence-based, personalized care by making sure that treatment aligns with what matters most to the patient, improving both satisfaction and results [1]. In addition, patient involvement in PROM design enhances relevance, acceptability, and completion rates.

PROMs can be used to classify patients based on their likelihood of benefiting from specific interventions. By analyzing PROM data alongside clinical data, it becomes possible to identify predictors of patient-centered success for various treatments. For example, a patient with a high baseline burden of chronic pain may respond better to certain approaches that focus on pain management. Incorporating PROMs into decision-making frameworks for treating benign surgical conditions allows for data-driven recommendations tailored to each patient, similar to the principles of precision medicine [38].

## 9. Equity, Generalizability, and Future Directions for PROs in Hernia Surgery

Beyond psychometric and implementation issues, a crucial but underexplored dimension of PRO research in hernia surgery concerns equity and generalizability. Most validated instruments and registries originate in high-income countries, often within highly digitized healthcare systems. As such, there is a risk that the voices of patients in low- and middle-income countries, or those outside large academic centers, remain underrepresented. Evidence shows that socioeconomic status, education level, and health literacy significantly influence both the likelihood of completing PRO instruments and the interpretation of reported outcomes [39,40]. Without explicit efforts to ensure inclusivity, PRO data may inadvertently reinforce disparities by primarily reflecting the experiences of patients already well-served by healthcare systems.

A related challenge is cross-cultural validity. Even when instruments are translated, linguistic equivalence does not guarantee conceptual equivalence. Terms such as “discomfort”, “activity limitation”, and “cosmetic concern” may carry different cultural meanings, and responses can be shaped by local expectations of surgical outcomes. This highlights the need for not only translation, but cultural adaptation and re-validation of PRO tools across contexts [41,42].

Looking ahead, future research should prioritize the development of internationally comparable yet contextually adaptable PRO frameworks. One promising approach is the use of modular instruments: a generic core set of questions combined with regionally tailored modules that address cultural or healthcare-specific concerns. Furthermore, embedding PRO collection within surgical registries could generate large, diverse datasets that improve both external validity and benchmarking.

Finally, equity in implementation will require pragmatic strategies to avoid digital exclusion. While electronic platforms facilitate real-time capture and integration with hospital records, they may marginalize older patients or those without reliable internet access. Hybrid models—combining digital, paper, and interviewer-administered surveys—remain necessary to ensure that PRO research reflects the full patient population undergoing hernia surgery.

## 10. Call to Action for Surgeons to Incorporate Patient-Centered Outcomes for Holistic Evaluations

However, integrating PROs is not without challenges. Time constraints, resource allocation for data collection, and the need for effective data analysis pose significant barriers. Still, the benefits outweigh these difficulties. PROs offer a nuanced understanding that can lead to more patient-centered care plans, better communication between patients and surgeons, and a stronger therapeutic bond. Additionally, these measures can highlight areas for quality improvement, support patient-focused care at the organizational level, and guide clinical research within the field to enhance outcomes [43].

Adopting PROs requires a multidisciplinary approach. For instance, linking reimbursement to PRO reporting may incentivize adoption. Training staff to administer and interpret these outcomes is important, as is having strong IT systems for data management. Feedback mechanisms, such as automated follow-up questionnaires, must be established to ensure PROs inform clinical decisions and policy-making. Proper use of PRO data can help healthcare professionals customize postoperative care to address common issues like pain management, emotional support, and functional rehabilitation, as well as manage expectations about recovery timelines.

Healthcare professionals must advocate for policy changes that mandate the inclusion of PROs in surgical evaluations. By promoting PROs at both the institutional and regulatory levels, healthcare providers can help ensure these metrics become standard, fostering a culture that emphasizes patient-centered care. The ultimate goal is to incorporate PROs into routine surgical assessments, thereby improving overall patient outcomes and satisfaction. Additionally, it is necessary to include PROs in core outcome sets for outcomes research in various benign surgical procedures.

## 11. Conclusions

PROs should become the default benchmark for evaluating outcomes in hernia surgery, complementing—not replacing—traditional measures. Incorporating PROs into surgical evaluations for benign conditions like hernia surgery is not only beneficial but arguably essential for delivering comprehensive care. The adoption of PROs shifts the focus from solely measuring clinical success to a broader assessment of patient well-being, recognizing the patient’s subjective experience as a key element of recovery. It is natural to monitor whether the patient’s primary concern leading to surgery improves afterward. In other words, it is essential to directly assess the patient’s primary concern and then track the outcome related to this problem post surgery. This shift in perspective can significantly improve the quality and effectiveness of surgical care, ensuring that patient recovery emphasizes not only preventing complications and recurrences but also achieving optimal well-being. Healthcare professionals stand at important crossroads where their active involvement and advocacy for integrating PROs can foster meaningful progress in patient-centered care.

## Data Availability

No new data were created or analyzed in this study. Data sharing is not applicable to this article.

## Abbreviations

The following abbreviations are used in this manuscript:

PROs	patient-reported outcomes
PROMs	patient-reported outcome measures
MYMOP	Measure Yourself Medical Outcome Profile
SF-36	Medical Outcomes Study 36-Item Short-Form Health Survey
WOMAC	Western Ontario and McMaster Universities Osteoarthritis Index
ICHOM	International Consortium for Health Outcomes Measurement
PROMIS	Patient-Reported Outcomes Measurement Information System
AI	artificial intelligence
VBHC	Value-Based HealthCare
KPIs	key performance indicators
PREMs	patient-reported experience measures
